# Perioperative ASL-derived CBF changes are associated with complications after Moyamoya bypass surgery

**DOI:** 10.3389/fneur.2026.1928443

**Published:** 2026-10-05

**Authors:** Jiachao Lu, Chao Li, Tiansheng Su, Yong Mo, Guangxiang Huang, Shuling Tang, Ligen Mo, Chaojue Huang, Jun Yan

**Affiliations:** 1 Department of Neurosurgery, Guangxi Medical University Cancer Hospital, Nanning, China; 2 Department of Neurosurgery, The First Affiliated Hospital of Guangxi Medical University, Nanning, China

**Keywords:** ASL, cerebral blood flow, extracranial-intracranial bypass, Moyamoya disease, postoperative complications

## Abstract

**Background:**

Postoperative hyperperfusion and insufficient perfusion augmentation are important contributors to morbidity after revascularization surgery for Moyamoya disease (MMD). Arterial spin labeling (ASL) enables noninvasive assessment of cerebral blood flow (CBF), but the relationship between perioperative ASL-derived CBF changes and specific postoperative complications after direct bypass remains incompletely defined. This study evaluated whether perioperative ASL-derived CBF changes were associated with postoperative complications after direct bypass surgery in patients with MMD.

**Methods:**

We retrospectively reviewed 146 patients with MMD who underwent first-time direct bypass surgery between June 2020 and October 2024. Preoperative ASL and a standardized postoperative ASL examination on postoperative day 5 were performed, and CBF values were measured in the recipient vascular territory. Subsequent intracranial hemorrhage, cerebral infarction, and postoperative seizures occurring within 90 days after surgery were analyzed. Firth penalized logistic regression was used for the hemorrhage outcome, conventional logistic regression was used for cerebral infarction, and ROC analyses included bootstrap assessment of cutoff uncertainty and 0.632 + bootstrap correction of AUC optimism.

**Results:**

Postoperative intracranial hemorrhage occurred in 8 patients (5.5%), cerebral infarction in 14 patients (9.6%), and postoperative seizures in 9 patients (6.2%); all events occurred after the day-5 ASL examination and within 90 days after surgery. ASL-derived CBF change was associated with postoperative intracranial hemorrhage in Firth penalized logistic regression (OR, 1.219; 95% profile-likelihood CI, 1.102–1.580; *p* < 0.001). The exploratory cutoff was >43.48 mL/100 g/min, with an apparent AUC of 0.9891 and a 0.632 + corrected AUC of 0.9890. ASL-derived CBF change was inversely associated with postoperative cerebral infarction (OR, 0.885; 95% CI, 0.839–0.933; *p* < 0.001). The exploratory cutoff was ≤4.48 mL/100 g/min, with an apparent AUC of 0.9150 and a 0.632 + corrected AUC of 0.9150. No significant association was observed between ASL-derived CBF change and postoperative seizures.

**Conclusion:**

Perioperative ASL-derived CBF change was associated with hemorrhagic and ischemic complications after direct bypass surgery for MMD. Marked postoperative CBF elevation was observed in patients with intracranial hemorrhage, whereas insufficient perfusion augmentation was associated with cerebral infarction. The proposed thresholds are exploratory, cohort-specific estimates and require external validation before clinical application.

## 1 Introduction

Moyamoya disease (MMD) is a progressive cerebrovascular disorder in which stenosis or occlusion gradually involves the terminal internal carotid arteries and the proximal anterior and middle cerebral arteries, leading to the formation of abnormal and fragile collateral vessels at the base of the brain (1, 2). In symptomatic MMD, revascularization surgery is widely accepted as the principal treatment because it restores cerebral blood supply and reduces the risk of subsequent ischemic or hemorrhagic episodes (3).

Direct extracranial-intracranial (EC-IC) bypass provides immediate flow augmentation (4); however, postoperative hemodynamic imbalance remains an important contributor to morbidity after revascularization (5). Excessive focal hyperperfusion after bypass may predispose patients to intracranial hemorrhage, whereas insufficient perfusion augmentation or postoperative hypoperfusion may increase the risk of cerebral infarction. Therefore, early identification of these opposing hemodynamic states is essential for individualized postoperative monitoring and management.

Arterial spin labeling (ASL) is a noninvasive magnetic resonance perfusion technique that uses magnetically labeled arterial blood water as an endogenous tracer and allows quantitative assessment of cerebral blood flow (CBF) without contrast administration (6). Given its safety and repeatability, ASL is well suited for perioperative monitoring in MMD (1, 7). Prior work has shown that ASL can detect postoperative cerebral perfusion changes and may help identify cerebral hyperperfusion syndrome after revascularization in patients with MMD (8). Conversely, ASL-derived regional CBF reduction, often accompanied by arterial transit artifacts, has been used to characterize hypoperfusion in ischemic MMD and to guide surgical-side selection (9). However, whether perioperative ASL-derived CBF changes can stratify specific hemorrhagic and ischemic complications after direct bypass surgery remains unclear.

In this retrospective study, we investigated whether perioperative ASL-derived perfusion changes were associated with postoperative intracranial hemorrhage, cerebral infarction, and seizures in patients with MMD undergoing direct EC–IC bypass. We further explored the apparent discriminatory performance of ASL-derived CBF change and derived cohort-specific thresholds for hemorrhagic and ischemic complications after surgery.

## 2 Materials and methods

### 2.1 Study design and patients

This retrospective study included patients diagnosed with Moyamoya disease (MMD) who received their first-time direct extracranial-intracranial (EC-IC) bypass surgery at the Department of Neurosurgery, First Affiliated Hospital of Guangxi Medical University, between June 2020 and October 2024. MMD was diagnosed using digital subtraction angiography (DSA) in accordance with the 2021 revised diagnostic criteria for Moyamoya disease (10).

The inclusion criteria were as follows: (1) age between 18 and 70 years; (2) diagnosis of MMD confirmed by DSA; (3) first-time direct EC-IC bypass surgery; (4) availability of both preoperative and postoperative arterial spin labeling (ASL) examinations; and (5) postoperative follow-up longer than 3 months. The exclusion criteria were as follows: (1) isolated cerebrovascular stenosis or occlusion without angiographic features of MMD; (2) previous revascularization surgery; (3) severe cognitive impairment precluding reliable clinical assessment; (4) severe cardiac, pulmonary, hepatic, or renal insufficiency; and (5) incomplete clinical, imaging, or follow-up data. Patients were identified retrospectively from the institutional surgical database. The analytic cohort comprised 146 patients who satisfied the prespecified eligibility criteria.

This study was approved by the Ethics Committee of the First Affiliated Hospital of Guangxi Medical University [approval number: 2026-E0227]. All patient data were anonymized before analysis.

### 2.2 Surgical procedure

All patients underwent preoperative DSA and perioperative ASL imaging. Direct EC-IC bypass was performed under general anesthesia using STA-MCA anastomosis, a standard direct revascularization procedure for MMD that can provide immediate flow augmentation to the affected MCA or recipient vascular territory (11). The distal STA parietal branch was prepared as the donor vessel and tunneled intracranially. After arachnoid opening, a suitable cortical M4 branch of the middle cerebral artery (MCA) was selected as the recipient vessel. End-to-side anastomosis between the STA parietal branch and the M4 segment was completed under microscopic visualization. FLOW800 confirmed bypass patency and recipient territory perfusion.

### 2.3 ASL assessment and follow-up

Patient demographic data, mRS scores, and perioperative ASL-derived CBF values were retrieved from the hospital information system. CBF measurements were obtained using ASL with a post-labeling delay (PLD) of 1.5 s. Postoperative ASL was performed on postoperative day 5 in all included patients. Regions of interest (ROIs) were manually placed on the ASL CBF maps within the MCA territory supplied by the STA–MCA bypass, predominantly in the temporal or frontal region according to the location of the recipient artery. Circular ROIs with a diameter of approximately 15 mm were used, while large cortical vessels, chronic infarcted areas, and regions with obvious imaging artifacts were avoided. Corresponding structural MR images were used as anatomical references to maintain consistent ROI locations between the preoperative and postoperative examinations. Three ROIs were measured within the bypass-supplied territory, and the mean value was used as the regional CBF value. CBF change was defined as postoperative CBF minus preoperative CBF.

Because a single PLD of 1.5 s was used, ASL-derived CBF measurements may be affected by prolonged arterial transit time (ATT) and arterial transit artifacts (ATA) in MMD. Standard-delay ASL may underestimate tissue CBF when labeled blood has not fully reached the tissue, whereas residual intravascular labeled signal may produce focal hyperintensity and overestimate apparent regional CBF. Accordingly, the measured values were interpreted as ASL-derived perfusion estimates rather than absolute measures of tissue perfusion, and preoperative-to-postoperative differences may partly reflect changes in ATT in addition to true changes in tissue perfusion (12).

Postoperative complications were defined as intracranial hemorrhage, cerebral infarction, or postoperative seizures occurring within 90 days after surgery. Postoperative intracranial hemorrhage and cerebral infarction were identified from postoperative CT and/or MRI findings together with the corresponding clinical records. Postoperative seizures were identified from documented clinical seizure events. ASL-derived CBF values were not used as criteria for outcome adjudication. All analyzed complication events occurred after the postoperative day-5 ASL examination. Separate comparisons were performed for each complication category versus patients without that complication. Because the eligibility criteria required more than 3 months of postoperative follow-up, the 90-day outcome window was available for all included patients.

### 2.4 Statistical analysis

Statistical analyses were performed using SPSS version 26.0 and R version 4.5.0. Normally distributed continuous variables are presented as mean ± standard deviation and were compared using the independent-samples *t*-test; non-normally distributed variables are presented as median (interquartile range) and were compared using the Mann–Whitney *U* test. Categorical variables are presented as *n* (%) and were compared using the chi-square test or Fisher’s exact test, as appropriate.

ASL-derived CBF change, defined as postoperative minus preoperative CBF, was the primary perfusion variable. Variables associated with each outcome at *p* < 0.05 in univariable analyses were considered candidate covariates. Because CBF change is algebraically derived from preoperative and postoperative CBF, these three perfusion measures were not entered simultaneously into the same regression model. For postoperative intracranial hemorrhage, Firth penalized logistic regression was used because only eight events occurred and conventional maximum-likelihood estimates were potentially unstable; ASL-derived CBF change and hypertension were included in the model. For cerebral infarction, conventional logistic regression was performed with ASL-derived CBF change as the predictor. Odds ratios (ORs) with 95% confidence intervals (CIs) were reported.

Receiver operating characteristic (ROC) analysis was used to assess the discriminatory performance of ASL-derived CBF change for intracranial hemorrhage and cerebral infarction. Optimal cutoffs were determined using the Youden index, and apparent AUCs with 95% CIs, sensitivity, specificity, positive predictive value, and negative predictive value were calculated. Stratified bootstrap resampling with 5,000 iterations was used to obtain percentile 95% CIs for the cutoff values. In addition, 0.632 + bootstrap correction with 5,000 resamples was used to estimate optimism-corrected AUCs. Both the apparent and internally corrected AUCs, as well as the derived thresholds, were considered exploratory and require independent external validation.

Given the three postoperative outcomes evaluated, a Bonferroni-adjusted significance threshold of *p* < 0.017 was additionally considered for outcome-specific inference; *p* < 0.05 was retained for descriptive and exploratory univariable analyses. No *a priori* sample-size calculation was performed because all eligible patients during the predefined study period were included. Because only 9 postoperative seizure events occurred, a fixed-sample simulation-based sensitivity analysis was additionally performed. ASL-derived CBF change was standardized to 1 SD, and the minimum detectable odds ratio (OR) was estimated for 80% power using a two-sided α of 0.017, with the observed sample size and event frequency held fixed. Analyses of outcomes with few events were therefore considered exploratory.

## 3 Results

### 3.1 Complication profile

A total of 146 patients with Moyamoya disease who underwent direct extracranial–intracranial bypass surgery were included in the final analysis. Within 90 days after surgery, intracranial hemorrhage occurred in 8 patients (5.5%), cerebral infarction in 14 patients (9.6%), and postoperative seizures in 9 patients (6.2%). Representative perioperative angiographic, intraoperative, FLOW800 fluorescence videoangiographic, and ASL perfusion findings are shown in Figure 1. Supplementary Figure S1 shows representative angiographic Suzuki stages.

**Figure 1:**
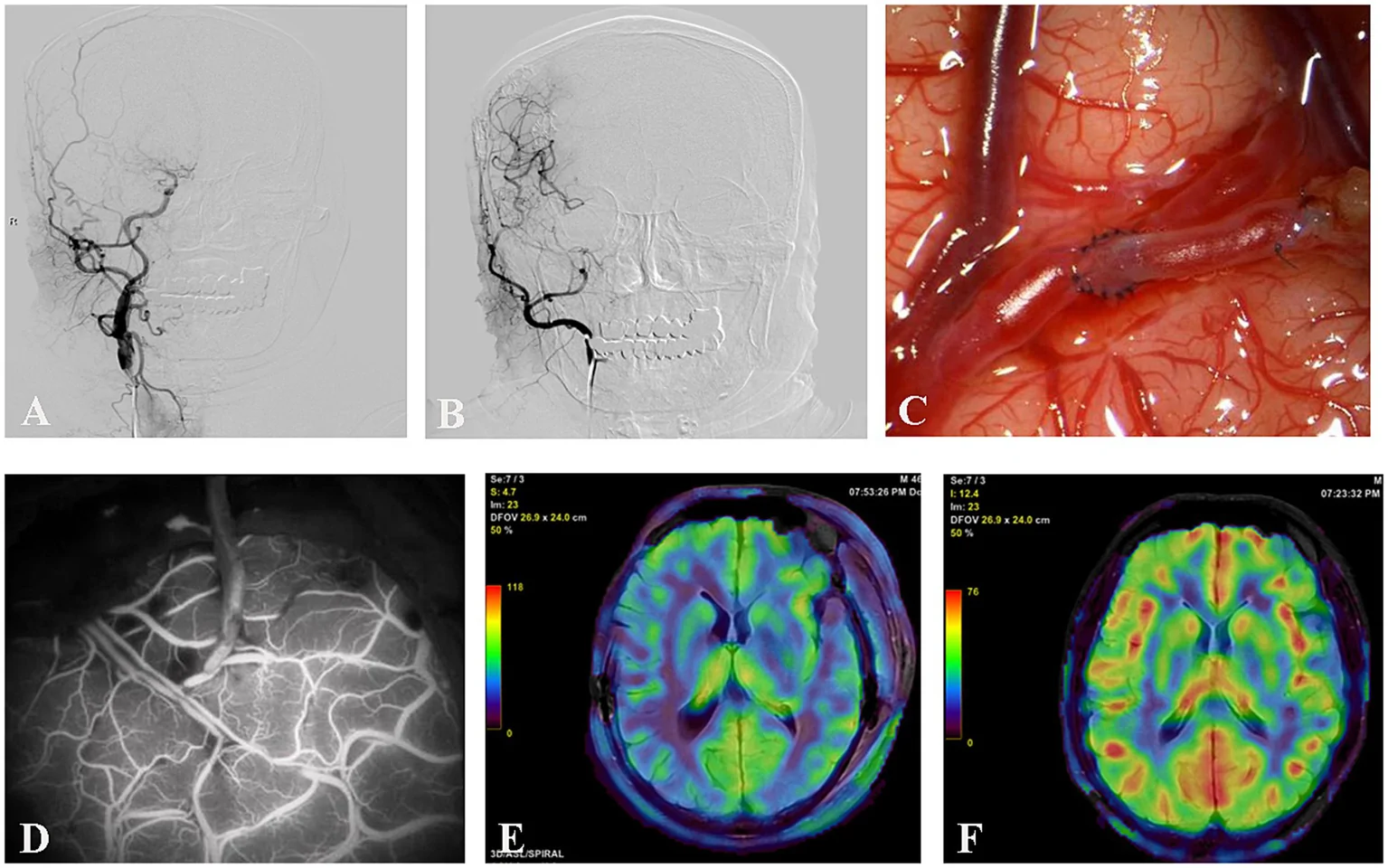
Representative perioperative imaging findings in a patient undergoing direct bypass for Moyamoya disease. **(A)** Preoperative angiography. **(B)** Postoperative angiography demonstrating a patent bypass and improved cortical perfusion. **(C)** Intraoperative view of the completed bypass. **(D)** FLOW800 fluorescence videoangiography confirming anastomotic patency. **(E)** Preoperative ASL perfusion image. **(F)** Postoperative ASL perfusion image demonstrating increased perfusion in the recipient territory.

### 3.2 ASL-derived CBF change and postoperative intracranial hemorrhage

Univariable analysis showed that hypertension history, postoperative ASL-derived CBF, and ASL-derived CBF change differed significantly between patients with and without postoperative intracranial hemorrhage (Table 1). Patients who developed postoperative intracranial hemorrhage had substantially higher postoperative ASL-derived CBF than those without hemorrhage (100.31 ± 24.16 vs. 55.06 ± 18.71 mL/100 g/min; *p* < 0.001). ASL-derived CBF change was also markedly greater in the hemorrhage group than in the non-hemorrhage group (61.78 ± 12.78 vs. 11.71 ± 18.20 mL/100 g/min; *p* < 0.001). Hypertension was more frequent among patients who developed postoperative hemorrhage (62.5% vs. 20.3%; *p* = 0.019). No significant between-group differences were observed for sex, age, initial symptoms, mRS score, Suzuki stage, preoperative ASL-derived CBF, or operated side.

**Table 1:** Univariable analysis of factors associated with postoperative intracranial hemorrhage.

Variable	Hemorrhage		t/Z/χ2 value	*p*-value
	Yes (*n* = 8)	No (*n* = 138)		
Gender			0.870	0.351
Male	6 (75.00)	71 (51.45)		
Female	2 (25.00)	67 (48.55)		
Age	43.62 ± 8.28	45.82 ± 11.08	0.550	0.583
Initial symptoms				
Headache/dizziness	2 (25.00)	28 (20.29)	0.000	>0.999
Hemorrhage	0 (0.00)	23 (16.67)	0.576	0.448
Infarction	2 (25.00)	37 (26.81)	0.000	>0.999
TIA	4 (50.00)	45 (32.61)	0.394	0.530
Epilepsy	0 (0.00)	6 (4.35)	–	>0.999
Hypertension history			5.477	0.019
YES	5 (62.50)	28 (20.29)		
NO	3 (37.50)	110 (79.71)		
mRS	1.00 (0.00–1.00)	1.00 (0.00–3.00)	0.941	0.346
Suzuki Stage	3.00 (2.75–3.00)	3.00 (2.00–4.00)	0.280	0.779
Preop ASL-CBF	38.53 ± 14.27	43.35 ± 20.18	0.664	0.508
Postop ASL-CBF	100.31 ± 24.16	55.06 ± 18.71	−6.545	<0.001
ASL CBF Change	61.78 ± 12.78	11.71 ± 18.20	−7.660	<0.001
Surgical side			0.000	>0.999
Right	4 (50.00)	78 (56.52)		
Left	4 (50.00)	60 (43.48)		

Values are shown as *n* (%), mean ± SD, or median (IQR), as appropriate. *t* values indicate independent-samples *t*-tests, *Z* values indicate Mann–Whitney *U* tests, and *χ^2^* values indicate chi-square tests. Fisher’s exact test was applied when expected cell counts were <5.

Given the limited number of hemorrhagic events (*n* = 8) and the instability of conventional maximum-likelihood estimates, Firth penalized logistic regression was used for the hemorrhage model. ASL-derived CBF change remained significantly associated with postoperative intracranial hemorrhage (OR, 1.219; 95% profile-likelihood CI, 1.102–1.580; *p* < 0.001; Table 2). The estimated association for hypertension was attenuated compared with that obtained using conventional logistic regression (OR, 19.979; 95% profile-likelihood CI, 1.491–1420.898; *p* = 0.022), but the confidence interval remained extremely wide. Moreover, hypertension did not meet the bonferroni-adjusted significance threshold of *p* < 0.017, indicating substantial statistical uncertainty surrounding this estimate (Table 2). A representative case of postoperative intracranial hemorrhage associated with marked postoperative hyperperfusion is shown in Figure 2.

**Table 2:** Firth penalized logistic regression analysis for postoperative intracranial hemorrhage.

Variable	OR	95% profile-likelihood CI	*p*-value
ASL CBF change	1.219	1.102–1.580	<0.001
Hypertension	19.979	1.491–1420.898	0.022

Firth penalized likelihood logistic regression was used because of the limited number of hemorrhagic events and potential sparse-data/separation bias. A bonferroni-adjusted significance threshold of *p* < 0.017 was considered for the three outcome-specific analyses.

**Figure 2:**
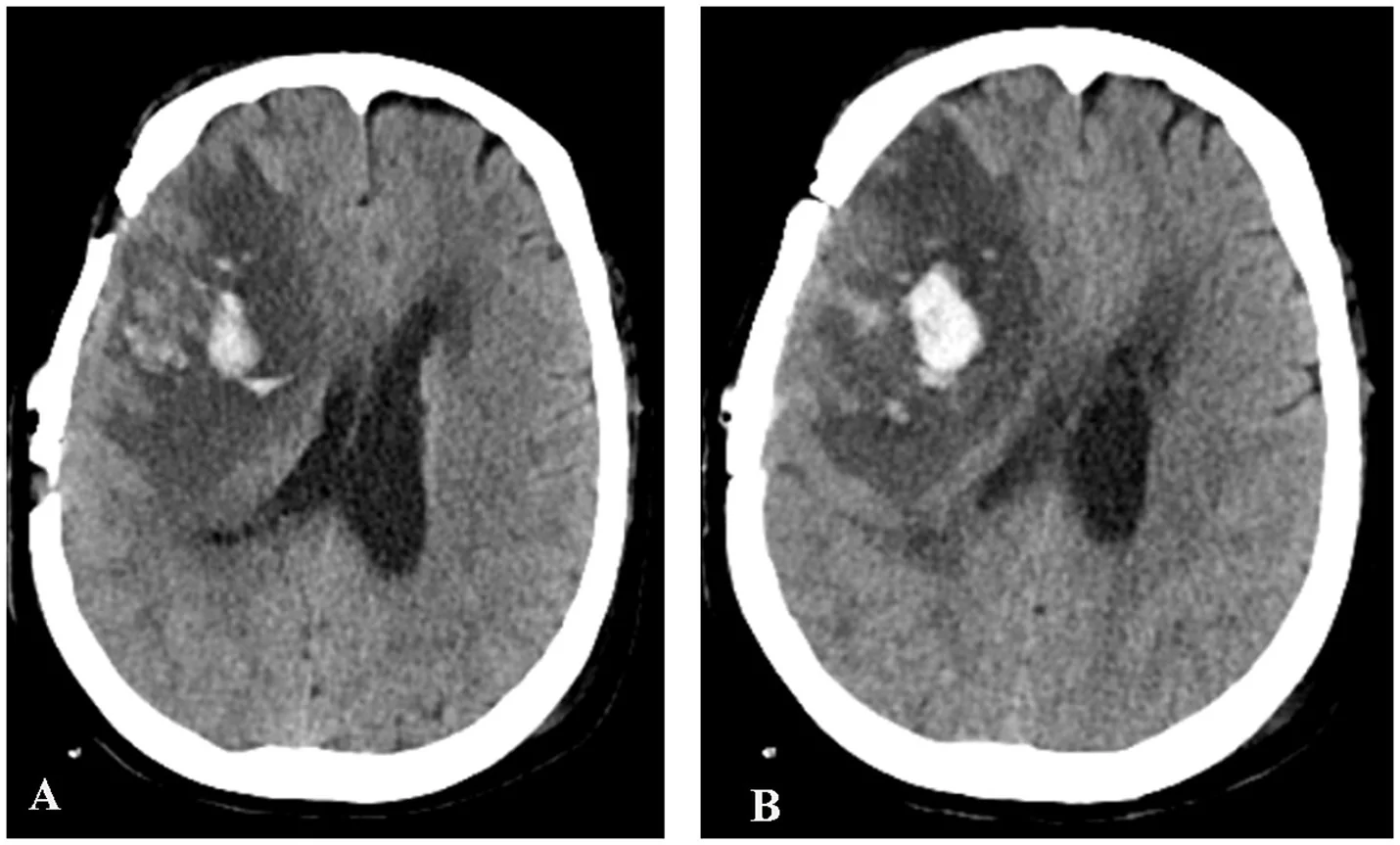
Representative postoperative imaging findings of intracranial hemorrhage after direct bypass surgery for Moyamoya disease. **(A,B)** Postoperative non-contrast computed tomography images showing intracranial hemorrhage in the right hemisphere after revascularization surgery.

### 3.3 ASL-derived CBF change and postoperative cerebral infarction

Univariable analysis showed significant differences in preoperative ASL-derived CBF, postoperative ASL-derived CBF, and ASL-derived CBF change between patients with and without postoperative cerebral infarction (Table 3). Patients who developed postoperative cerebral infarction had higher preoperative ASL-derived CBF than those without infarction (60.67 ± 23.29 vs. 41.22 ± 18.65 mL/100 g/min; *p* < 0.001), but lower postoperative ASL-derived CBF (45.79 ± 21.67 vs. 58.79 ± 21.28 mL/100 g/min; *p* = 0.032). ASL-derived CBF change was substantially lower in the infarction group than in the non-infarction group (−14.88 ± 16.02 vs. 17.57 ± 19.30 mL/100 g/min; *p* < 0.001), indicating insufficient postoperative perfusion augmentation or postoperative hypoperfusion in patients who developed infarction. Sex, age, initial symptoms, hypertension history, mRS score, Suzuki stage, and operated side were not significantly different between the two groups.

**Table 3:** Univariable analysis of factors associated with postoperative cerebral infarction.

Variable	Infarction		*t*/*Z*/*χ2* value	*p*-value
	Yes (*n* = 14)	No (*n* = 132)		
Gender			0.047	0.829
Male	7 (50.00)	70 (53.03)		
Female	7 (50.00)	62 (46.97)		
Age	44.00 ± 13.34	45.88 ± 10.70	0.610	0.543
Initial symptoms				
Headache/dizziness	2 (14.29)	28 (21.21)	0.069	0.793
Hemorrhage	2 (14.29)	21 (15.91)	0.000	>0.999
Infarction	5 (35.71)	34 (25.76)	0.233	0.629
TIA	5 (35.71)	44 (33.33)	0.000	>0.999
Epilepsy	0 (0.00)	6 (4.55)	–	>0.999
Hypertension history			2.464	0.117
YES	6 (42.86)	27 (20.45)		
NO	8 (57.14)	105 (79.55)		
mRS	1.00 (0.00–2.75)	1.00 (0.00–2.25)	0.511	0.609
Suzuki stage	3.00 (2.00–3.00)	3.00 (2.00–4.00)	1.891	0.059
Preop ASL-CBF	60.67 ± 23.29	41.22 ± 18.65	−3.621	<0.001
Postop ASL-CBF	45.79 ± 21.67	58.79 ± 21.28	2.170	0.032
ASL CBF change	−14.88 ± 16.02	17.57 ± 19.30	6.066	<0.001
Surgical side			1.114	0.291
Right	6 (42.86)	76 (57.58)		
Left	8 (57.14)	56 (42.42)		

Values are shown as *n* (%), mean ± SD, or median (IQR), as appropriate. *t* values indicate independent-samples *t*-tests, *Z* values indicate Mann–Whitney *U* tests, and *χ^2^* values indicate chi-square tests. Fisher’s exact test was applied when expected cell counts were <5.

In logistic regression analysis, ASL-derived CBF change was inversely associated with postoperative cerebral infarction (OR, 0.885; 95% CI, 0.839–0.933; *p* < 0.001) (Table 4). This finding indicates that greater postoperative CBF augmentation was associated with lower odds of postoperative cerebral infarction. A representative case of postoperative cerebral infarction is shown in Figure 3.

**Table 4:** Logistic regression analysis of ASL-derived CBF change for postoperative cerebral infarction.

Variable	Coefficient	Std. Error	Wald *χ2*	*p*-value	OR (95%CI)
ASL CBF change	−0.122	0.027	20.223	<0.001	0.885(0.839–0.933)
Constant	−2.206	0.408	29.219	<0.001	–

**Figure 3:**
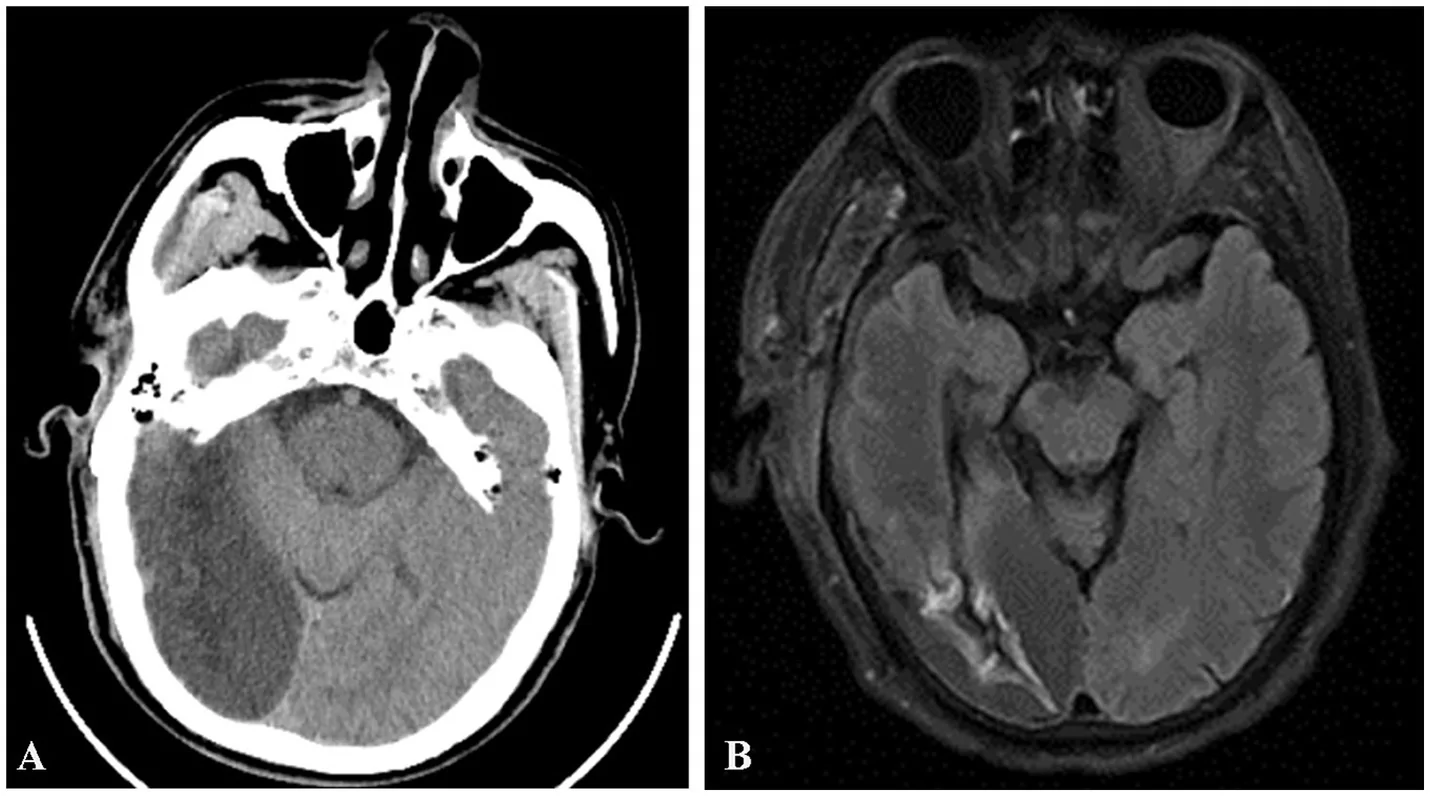
Representative postoperative imaging findings of cerebral infarction after direct bypass surgery for Moyamoya disease. **(A)** Postoperative computed tomography image showing a low-density lesion in the right temporal region after revascularization surgery. **(B)** Postoperative magnetic resonance image showing the corresponding ischemic lesion in the right temporal lobe.

### 3.4 Discriminatory performance of ASL-derived CBF change

ROC analysis demonstrated strong apparent discrimination of ASL-derived CBF change for postoperative intracranial hemorrhage and cerebral infarction (Table 5; Figure 4). For postoperative intracranial hemorrhage, the apparent AUC was 0.9891 (95% CI, 0.956–0.999), and the 0.632 + bootstrap-corrected AUC was 0.9890; the Youden-derived cutoff was >43.48 mL/100 g/min (Figure 4A). For postoperative cerebral infarction, the apparent AUC was 0.9150 (95% CI, 0.857–0.955), and the 0.632 + bootstrap-corrected AUC was 0.9150; the Youden-derived cutoff was ≤4.48 mL/100 g/min (Figure 4B). Bootstrap percentile 95% CIs for the cutoff values were 39.61–49.39 mL/100 g/min for hemorrhage and −10.19–11.32 mL/100 g/min for infarction.

**Table 5:** Apparent and bootstrap-corrected discriminatory performance and exploratory cutoff values of ASL-derived CBF change for postoperative intracranial hemorrhage and cerebral infarction.

Outcome	Cut-off (mL/100 g/min)	Bootstrap 95% CI for cutoff	Apparent AUC (95% CI)	0.632 + corrected AUC	Sensitivity (%)	Specificity (%)	PPV (%)	NPV (%)
Intracranial hemorrhage	>43.48	39.61–49.39	0.989 (0.956–0.999)	0.989	100.0	95.65	57.1	100.0
Cerebral infarction	≤4.48	−10.19–11.32	0.915 (0.857–0.955)	0.915	92.86	78.79	31.7	99.0

**Figure 4:**
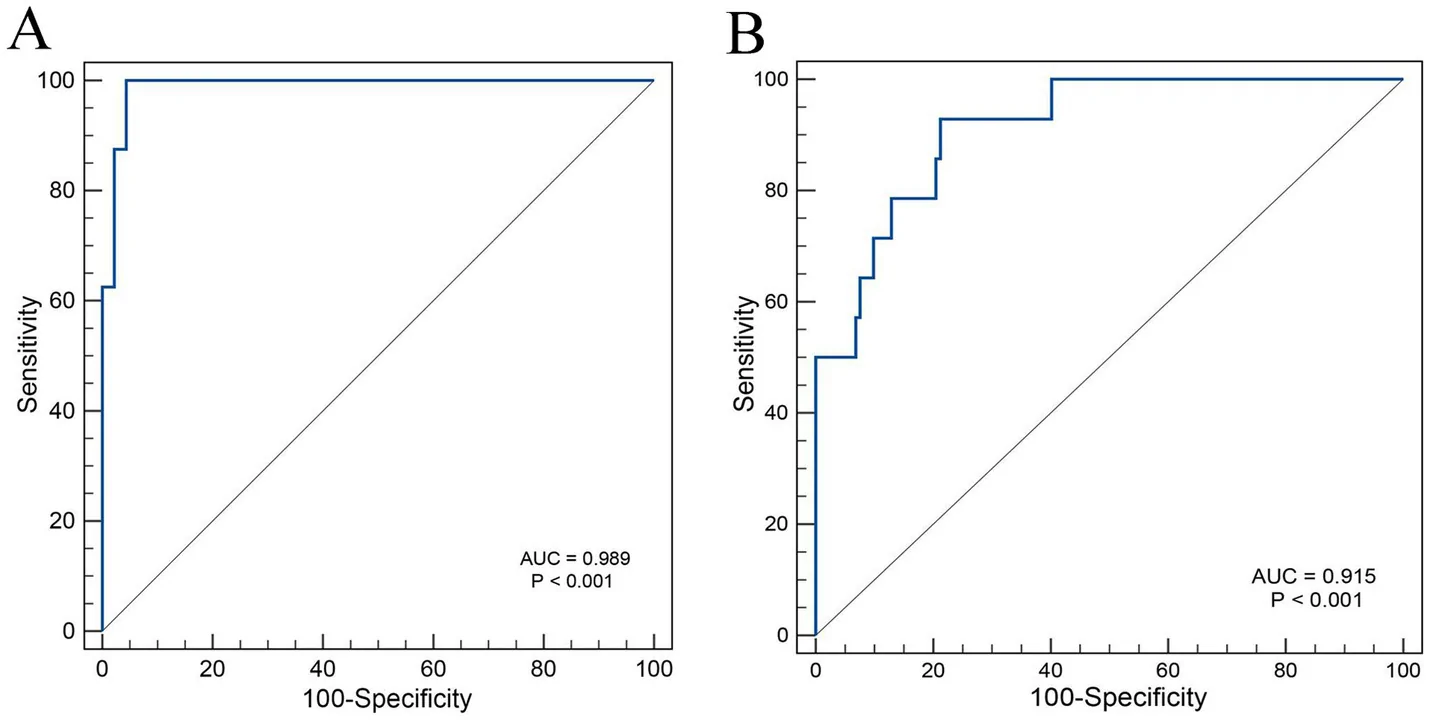
Receiver operating characteristic curves of ASL-derived CBF change for postoperative intracranial hemorrhage and cerebral infarction. **(A)** ROC curve of ASL-derived CBF change for identifying postoperative intracranial hemorrhage. **(B)** ROC curve of ASL-derived CBF change for identifying postoperative cerebral infarction.

Cutoff-based stratification showed marked differences in event rates. Postoperative intracranial hemorrhage occurred in 8 of 14 patients (57.1%) with an ASL-derived CBF change >43.48 mL/100 g/min, compared with 0 of 132 patients (0%) at or below this threshold (Fisher’s exact *p* = 7.13 × 10–10). Postoperative cerebral infarction occurred in 13 of 41 patients (31.7%) with an ASL-derived CBF change ≤4.48 mL/100 g/min, compared with 1 of 105 patients (1.0%) above this threshold (Fisher’s exact *p* = 1.56 × 10–7).

The 0.632 + corrected AUCs were nearly identical to the apparent AUCs, indicating minimal estimated optimism for discrimination based on this single prespecified continuous perfusion variable. Nevertheless, given the limited numbers of outcome events, particularly for intracranial hemorrhage, the AUCs and Youden-derived thresholds should be regarded as exploratory, cohort-specific estimates rather than established clinical decision thresholds. Internal bootstrap correction does not substitute for independent external validation.

### 3.5 ASL-derived CBF change and postoperative seizures

No demographic, clinical, or perfusion-related variable was significantly associated with postoperative seizures in the univariable analysis (Supplementary Table S1). In a univariable logistic model using standardized CBF change, the OR per 1-SD increase was 0.75 (95% CI, 0.37–1.52; *p* = 0.427). The fixed-sample sensitivity analysis showed that, with 146 patients, 9 seizure events, 80% power, and a two-sided α of 0.017, the available sample could reliably detect only relatively large associations, corresponding approximately to an OR ≥2.85 or ≤0.26 per 1-SD difference in ASL-derived CBF change. Thus, the study had limited ability to detect small-to-moderate perfusion–seizure associations, and no ASL-derived CBF cutoff was derived for postoperative seizures.

## 4 Discussion

In this retrospective cohort, perioperative ASL-derived CBF changes after STA–MCA bypass were associated with distinct postoperative complications in patients with MMD. Intraoperative FLOW800 confirmed immediate filling of the recipient artery and provided real-time evidence of bypass patency, whereas postoperative ASL demonstrated heterogeneous tissue-level perfusion responses after revascularization. Marked postoperative CBF elevation was observed in patients with intracranial hemorrhage, whereas reduced or insufficient CBF augmentation was observed in patients with cerebral infarction. In contrast, no significant association was identified between ASL-derived CBF change and postoperative seizures. These findings suggest that postoperative complications after EC–IC bypass may be associated with distinct hemodynamic patterns and support the potential value of ASL as an adjunct for postoperative perfusion assessment beyond confirmation of technical bypass patency.

Revascularization remains the cornerstone of surgical treatment for MMD, as the disease is characterized by progressive stenosis or occlusion of the terminal internal carotid arteries and their proximal branches, leading to chronic cerebral hypoperfusion and impaired cerebrovascular reserve (3). The primary objective of bypass surgery is to restore or augment cerebral perfusion by establishing an alternative extracranial-to-intracranial blood supply. Nevertheless, the postoperative hemodynamic response is not necessarily linear or uniformly beneficial. An excessive increase in perfusion may result in hyperperfusion-related injury, whereas insufficient perfusion augmentation may leave vulnerable brain tissue exposed to persistent or recurrent ischemia. Therefore, postoperative complications should not be interpreted solely from the perspective of technical patency, but rather in the broader context of regional perfusion balance and autoregulatory capacity.

In the present cohort, postoperative cerebral hemorrhage was significantly associated with a substantial increase in CBF within the bypass territory. This finding is pathophysiologically plausible. In chronically hypoperfused brain regions, long-standing vasodilation and impaired cerebrovascular autoregulation may reduce the ability of the microvascular bed to accommodate an abrupt increase in inflow after direct revascularization. Such a mismatch between restored arterial inflow and impaired distal vascular regulation may predispose fragile collateral vessels or microvasculature to hyperperfusion-related injury and hemorrhagic complications (13–15). This interpretation is consistent with previous ASL-based studies showing that postoperative perfusion MRI can detect hyperperfusion changes after revascularization in MMD, supporting the feasibility of ASL as a noninvasive tool for perioperative hemodynamic surveillance (8). Importantly, our findings further suggest that the magnitude and direction of postoperative CBF change, rather than bypass patency alone, may be clinically informative for identifying patients at risk of hemorrhagic events.

Direct comparison of perfusion thresholds across studies requires caution because published studies have used different imaging modalities, normalization strategies, time points, and outcome definitions. In our hemorrhage group, mean ASL-derived CBF increased from 38.53 to 100.31 mL/100 g/min, corresponding to a 160.3% relative increase and a postoperative/preoperative ratio of approximately 260.3%. Using IMP-SPECT, Kameyama et al. (16) reported postoperative/preoperative CBF ratio thresholds of 184.5% for local cerebral hyperperfusion and 241.3% for hemorrhagic hyperperfusion. Ishikawa et al. (17) reported hemorrhagic cerebral hyperperfusion syndrome with an rCBF increase of ≥30% using an operated-to-unoperated comparison and ≥50% using a postoperative-to-preoperative comparison. Intraoperative approaches have yielded different scales: an ICG-FLOW800 ΔCBF cutoff of 95.04% showed an AUC of 0.859 for cerebral hyperperfusion syndrome (18), whereas laser speckle contrast imaging identified an LΔCBF cutoff of 1.985 with an AUC of 0.726 (19). Our absolute cutoff of >43.48 mL/100 g/min cannot be directly converted into these ratio-based thresholds at the individual-patient level because baseline CBF varies. Nevertheless, the concordant direction of effect—marked flow augmentation accompanying hemorrhagic hyperperfusion—supports biological plausibility, while the numerical differences underscore the need for modality- and protocol-specific validation.

Postoperative cerebral infarction represents another major adverse event after bypass surgery for MMD (20). In our study, new postoperative infarction was significantly associated with a decrease in CBF within the corresponding vascular territory. This observation supports the concept that inadequate tissue perfusion remains a key mechanism of early postoperative ischemic injury. Although direct bypass is designed to improve cerebral perfusion, several perioperative factors may compromise effective flow delivery, including insufficient donor flow, transient hypotension or hypovolemia, competitive or redistributed flow after bypass, vasospasm, local thrombotic events, or early anastomotic dysfunction (21–24). Previous studies have also indicated that the degree of postoperative hemodynamic improvement is closely related to neurological recovery and clinical outcome in patients with MMD (25). Therefore, a patent bypass observed intraoperatively does not necessarily guarantee sufficient perfusion at the tissue level. In this context, postoperative ASL may complement intraoperative FLOW800 by identifying patients in whom regional cerebral perfusion remains inadequate despite technically successful anastomosis.

The higher preoperative ASL-derived CBF observed in patients who subsequently developed infarction appears counterintuitive and should not be interpreted as evidence of superior baseline tissue perfusion. In MMD, prolonged and heterogeneous ATT can distort single-PLD ASL in opposing directions. Delayed delivery of labeled blood can underestimate tissue CBF, while persistent intravascular labeled signal or ATA can produce focal hyperintensity and overestimate apparent regional CBF (12). Postoperative shortening of ATT can further alter the measured preoperative-to-postoperative difference. Recently infarcted tissue with luxury perfusion is another possible explanation in selected patients, although the present retrospective dataset cannot distinguish this mechanism. Because the archived ASL studies were not systematically rescored for ATA, the paradoxically high preoperative value should be regarded as a potential transit-related measurement phenomenon, and the CBF-change metric may contain an ATT component in addition to true tissue-perfusion change.

The contrasting associations observed for hemorrhage and infarction highlight the dual hemodynamic risk after direct revascularization in MMD. Excessive postoperative perfusion may increase the risk of hemorrhagic injury, whereas reduced or insufficient perfusion may predispose to ischemic events. This bidirectional pattern suggests that perioperative management should not only focus on confirming bypass patency, but also on detecting postoperative perfusion imbalance. ASL may be particularly useful in this setting because it provides quantitative or semi-quantitative CBF information without ionizing radiation or exogenous contrast agents. Compared with intraoperative visualization techniques, ASL has the potential to reflect postoperative tissue-level perfusion status and may therefore help guide individualized monitoring, blood pressure control, hydration management, and early intervention strategies. However, the clinical implementation of ASL-guided management requires further validation, particularly with respect to optimal timing, reproducibility, and clinically meaningful CBF thresholds.

No statistically significant association was identified between postoperative CBF changes and postoperative seizures in this study. The observed postoperative seizure incidence of 6.2% lies within the 95% CI of a recent proportional meta-analysis of adult direct revascularization, which reported a pooled postoperative seizure incidence of 3.99% (95% CI, 1.71–7.15%) (26). Postoperative seizures after revascularization are likely multifactorial and may be influenced by cortical irritation, local edema, blood–brain barrier disturbance, metabolic changes, hemorrhagic or ischemic lesions, recipient cortical location, hyperperfusion, or pre-existing epileptogenic susceptibility rather than by perfusion alteration alone (27–29). In addition, ASL-derived regional CBF change may not capture cortical excitability or microstructural injury relevant to seizure generation. With only 9 seizure events, the present analysis had limited precision and should be regarded as underpowered for moderate associations; therefore, the null finding does not exclude a clinically relevant relationship.

Several limitations should be acknowledged. First, this was a retrospective single-center study, with potential selection bias and limited generalizability. A complete pre-eligibility screening log was not retained, preventing reconstruction of the exact exclusion cascade. Second, the numbers of hemorrhagic and seizure events were small. Firth penalization reduced small-sample and separation bias in the hemorrhage model but cannot eliminate sparse-data uncertainty, as illustrated by the extremely wide confidence interval for hypertension. The seizure sensitivity analysis likewise showed limited ability to detect small-to-moderate associations. Third, although the 0.632 + bootstrap-corrected AUCs were nearly identical to the apparent AUCs, the high discrimination estimates and Youden-derived thresholds remain cohort-specific and potentially sensitive to the small number of events; internal bootstrap correction does not replace independent external validation. Fourth, ASL was acquired using a single PLD of 1.5 s. Prolonged and heterogeneous ATT, ATA, and postoperative shortening of transit time may affect both absolute CBF and the calculated preoperative-to-postoperative change. The retrospective ASL images were not systematically rescored for ATA, which limits interpretation of the unexpectedly higher preoperative ASL-derived CBF in the infarction group. Fifth, detailed scanner-level acquisition metadata beyond PLD and formal inter-rater or intra-rater reliability estimates were not available for the complete cohort, limiting assessment of measurement reproducibility. Sixth, potentially relevant perioperative confounders, including detailed PCA involvement, quantitative bypass graft flow, intraoperative hypotension, standardized postoperative blood pressure targets, antiplatelet or anticoagulant exposure, and fluid management, were not available with sufficient completeness for multivariable adjustment. Although postoperative ASL was standardized to day 5 and all analyzed outcomes occurred thereafter within a uniform 90-day window, exact event latency was not modeled. These limitations support treating the present findings as hypothesis-generating and warrant prospective multicenter validation using standardized ASL protocols, reproducibility assessment, comprehensive perioperative covariates, and prospective recording of event timing.

In summary, perioperative ASL-derived CBF change was associated with distinct postoperative hemodynamic phenotypes after EC–IC bypass for MMD: marked CBF elevation accompanied hemorrhagic complications, whereas insufficient perfusion augmentation accompanied cerebral infarction. These findings support the potential value of ASL as an adjunct for postoperative hemodynamic assessment, but the magnitude of the observed associations and the proposed thresholds should be interpreted in the context of small event numbers, single-PLD acquisition, residual confounding, and lack of external validation. Prospective multicenter studies using standardized ASL protocols and prespecified imaging time points are required before CBF-based thresholds can be used for clinical decision-making.

## 5 Conclusion

Perioperative ASL-derived CBF change was associated with hemorrhagic and ischemic complications after direct bypass surgery for MMD. Marked postoperative CBF elevation was observed in patients with intracranial hemorrhage, whereas insufficient perfusion augmentation was associated with cerebral infarction. The identified CBF-change thresholds should be considered exploratory, cohort-specific estimates and require external validation before clinical application.

## Data Availability

The raw data supporting the conclusions of this article will be made available by the authors, without undue reservation.

## Supplementary material

SUPPLEMENTARY FIGURE S1. Representative angiographic images illustrating different Suzuki stages in patients with Moyamoya disease.

SUPPLEMENTARY TABLE S1. Comparison of variables between patients with and without postoperative seizures.
