# Supplementary material for: Perioperative ASL-derived CBF changes are associated with complications after Moyamoya bypass surgery
Source: Front Neurol. 2026 Oct 5;17:1928443. doi: 10.3389/fneur.2026.1928443 (PMC13677357; doi:10.3389/fneur.2026.1928443)
Supplement: Supplementary file 1 [file Table_1.DOCX]

**Supplemental Table S1. Comparison of variables between patients with and without postoperative seizures**

| **Variables** | postoperative seizures | | t/Z/χ2 value | *P*-value |
| --- | --- | --- | --- | --- |
|  | Yes(n=9) | No(n=137) |  |  |
| **Gender** |  |  |  | 0.736 |
| Male | 4 (44.44) | 73 (53.28) | — |  |
| Female | 5 (55.56) | 64 (46.72) | — |  |
| Age | 38.89 ± 13.44 | 46.15 ± 10.66 | 1.947 | 0.054 |
| **Initial symptoms** |  |  |  |  |
| Headache/Dizziness | 3 (33.33) | 27 (19.71) | — | 0.391 |
| Hemorrhage | 0 (0.00) | 23 (16.79) | — | 0.355 |
| Infarction | 3 (33.33) | 36 (26.28) | — | 0.701 |
| TIA | 3 (33.33) | 46 (33.58) | — | >0.999 |
| Epilepsy | 0 (0.00) | 6 (4.38) | — | >0.999 |
| **Hypertension History** |  |  |  | >0.999 |
| YES | 2 (22.22) | 31 (22.63) | — |  |
| NO | 7 (77.78) | 106 (77.37) | — |  |
| mRS | 1.00 (1.00–2.00) | 1.00 (0.00–3.00) | 0.050 | 0.960 |
| Suzuki Stage | 3.00 (3.00–5.00) | 3.00 (2.00–4.00) | −1.288 | 0.198 |
| Preop ASL-CBF | 47.98 ± 20.01 | 42.76 ± 19.92 | −0.761 | 0.448 |
| Postop ASL-CBF | 57.00 ± 26.32 | 57.58 ± 21.35 | 0.077 | 0.939 |
| ASL CBF Change | 9.02 ± 24.27 | 14.81 ± 21.09 | 0.791 | 0.430 |
| **Surgical side** |  |  |  | 0.078 |
| Right | 8 (88.89) | 74 (54.01) | — |  |
| Left | 1 (11.11) | 63 (45.99) | — |  |

**Note:** Values are presented as n (%), mean ± SD, or median (IQR), as appropriate. Continuous variables were compared using the independent-samples t test or Mann–Whitney U test, as appropriate. Categorical variables were compared using Fisher’s exact test because of the small number of postoperative seizure events and expected cell counts <5; therefore, no χ² statistic is reported for these comparisons. Initial clinical presentations were not mutually exclusive. ASL, arterial spin labeling; CBF, cerebral blood flow; mRS, modified Rankin Scale; TIA, transient ischemic attack.
